# A Rare Case of Varicella-Zoster Virus Reactivation Following Recovery From COVID-19

**DOI:** 10.7759/cureus.12423

**Published:** 2021-01-01

**Authors:** Hardik D Desai, Kamal Sharma, Jaimini V Patoliya, Elton Ahadov, Neel N Patel

**Affiliations:** 1 Graduate Medical Education, Gujarat Adani Institute of Medical Sciences, Bhuj, IND; 2 Department of Cardiology, U.N. Mehta Institute of Cardiology and Research Center, BJ Medical College, Ahmedabad, IND; 3 Department of Biochemistry, Gujarat University, Ahmedabad, IND; 4 Graduate Medical Education, Azerbaijan Medical University, Baku, AZE; 5 Graduate Medical Education, BJ Medical College, Ahmedabad, IND

**Keywords:** herpes zoster virus, covid-19, sars-cov-2 (severe acute respiratory syndrome coronavirus -2), varicella-zoster virus

## Abstract

In patients with coronavirus disease 2019 (COVID-19), various cutaneous symptoms have been observed. Herpes zoster (HZ) is an infectious skin disease caused by the varicella-zoster virus (VZV) that, after a primary chickenpox infection, persists dormant in the dorsal root ganglia of cutaneous nerves. Unusual prolonged dermatological symptoms from recovered COVID-19 patients have rarely been recorded. In this report, we describe a case of HZ following recovery from COVID-19.

## Introduction

As of December 23, 2020, severe acute respiratory syndrome coronavirus 2 (SARS-CoV-2) has infected 76,382,044 people with 1,702,128 mortality worldwide as per World Health Organization (WHO) coronavirus disease 2019 (COVID-19) report. Emerging evidence suggests that it affects multiple organs with myriad clinical signs and symptoms. Preliminary evidence suggests that the virus also affects skin, nails, and mucus membranes, causing various dermatological manifestations. The majority of studies have reported reactivation of herpes zoster (HZ) during the acute/subacute phase of COVID-19. A wide variety of skin symptoms including maculopapular eruptions, morbilliform rashes, urticaria, chickenpox-like lesions, livedo reticularis, COVID toes, erythema multiforme, pityriasis rosea, and several other trends have been associated with COVID-19 [1].

## Case presentation

A 62-year-old Asian Indian vegan woman, known case of hypertension, non-diabetic with history of varicella-zoster virus (VZV) infection in childhood at nine years of age presented to the dermatology clinic with a chief complaint of painful blisters with ulcerations in few skin lesions and a complaint of burning sensation and mild itching over the right lower abdomen in an iliac region corresponding to right-sided T11-12 dermatome. The patient had a recent past medical history of COVID-19 which was diagnosed through nasal swab reverse transcriptase-polymerase chain reaction (RT-PCR). She was successfully treated with a home-based regimen of hydroxychloroquine, azithromycin, amoxycillin with clavulanic acid, and zinc sulfate with other supportive treatment and recovered after two weeks from COVID-19 diagnosis. The patient reported rashes that appeared in the form of fluid-filled bubbles that rupture upon scratching, releasing clear discharges over the right lower abdomen around the 20th day following the clinical recovery from COVID-19. Vital signs and physical examination were normal except for vesicles with surrounding erythema on the right lumbar and iliac region, unilaterally characteristically restricted to T11-T12 dermatomes, which made a clinical diagnosis of HZ. Laboratory blood tests were within a normal range including the inflammatory markers of COVID-19 infection as shown in Table.1.

**Table 1:** Clinical and laboratory characteristics in patient presenting with herpes zoster TLC: total leucocyte count, HCQ: hydroxychloroquine, g/DL: gram/decilitre, N/L ratio: neutrophil/lymphocyte ratio, CRP: C-reactive protein

Parameters	During COVID-19 illness (Day 14)	At the time of onset of Lesions (Day 20 - following the recovery of COVID-19)	After 10 days of cutaneous symptoms onset (Day 30- following the recovery of COVID-19)	After 20 days of cutaneous symptoms onset (Day 40-following the recovery of COVID-19)
Hemoglobin (g/dL)	10.1	10.4	11	-
TLC (thou/mm3)	6.5	9.8	10.41	-
Neutrophils (%)	73	64	46.30	-
Lymphocyte (%)	21	30	49.20	-
N/L ratio (%)	3.47	2.13	0.94	-
CRP (mg/L)	91.22	4.1	1.94	-
Ferritin ng/mL	178.8	-	-	-
Symptoms/Signs	High-grade Fever, Nonproductive Cough, Fatigue (Resolved on day 10)	Painful Red blister rash unilateral, Burning Sensation, Itching, No fever	Abdominal pain suggestive of Post-herpetic Neuralgia	Heaviness at the upper right quadrant of the abdomen
Imaging	C-Xray suggestive of Patchy infiltration	-	-	Cardiac and Abdominal USG non-conclusive
Treatment	Hydroxychloroquine, Azithromycin, Amoxicillin and Clavulanate, other supportive treatment	Acyclovir Oral, Acyclovir Topical, Supportive treatment	Pregabalin and Amitriptyline	Continued Pregabalin and Amitriptyline

**Figure 1:**
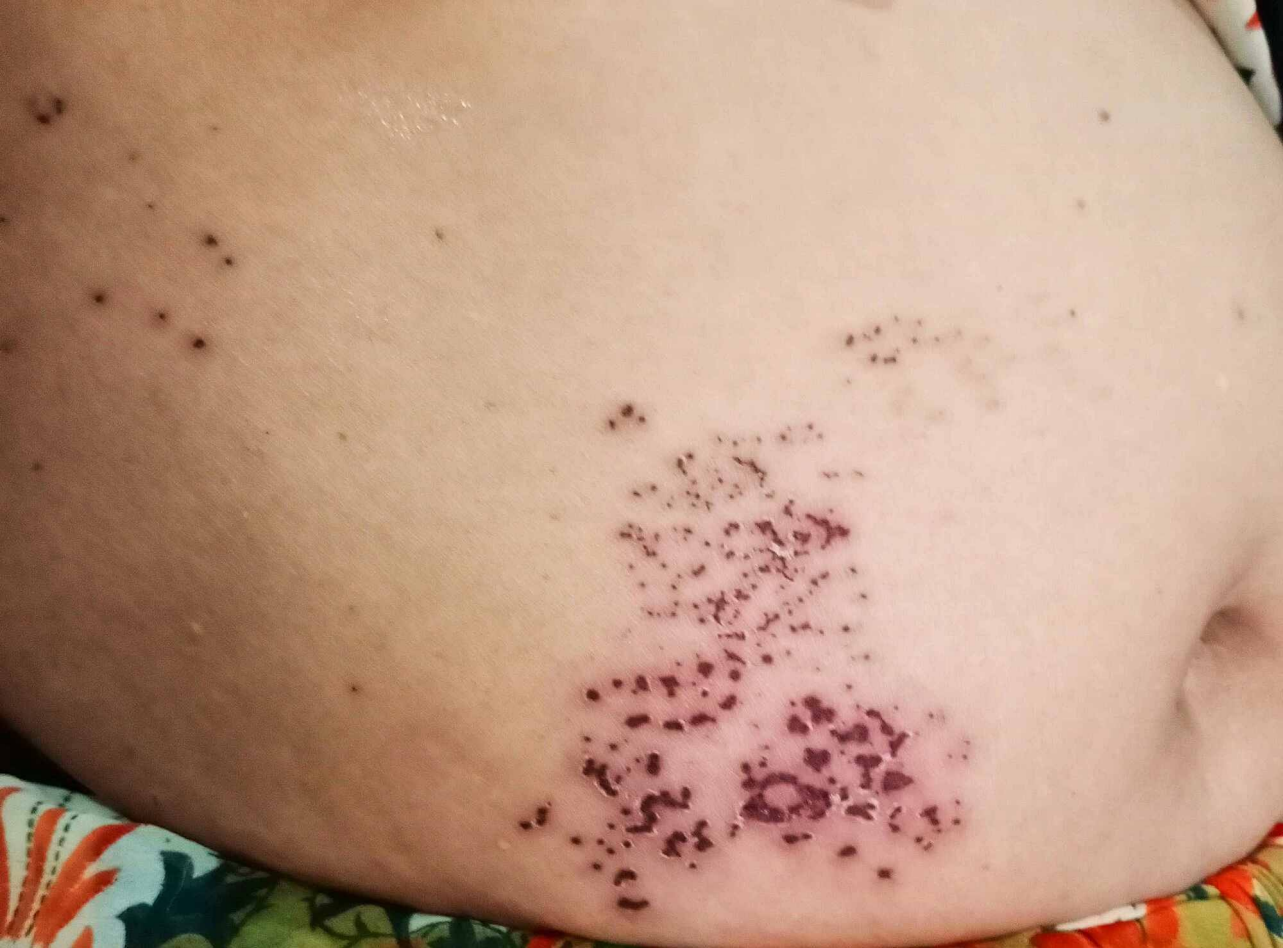
Showing skin lesion with crusting and vesiculation and surrounding erythema affecting T11-T12 dermatome.

She was treated with acyclovir 800 mg five times a day orally and along with topical application 5% acyclovir with other supportive symptomatic treatment for eight days. On the eighth day follow-up, she presented with a new onset symptom of superficial neuralgic pain suggestive of postherpetic neuralgia with a resolution of skin lesion as shown in (Figure 1).

Laboratory test on follow up showed lymphocytosis suggestive of viral infection with a negative inflammatory marker. She was treated with 75 mg/day of pregabalin and 25mg/day of amitriptyline orally for two weeks. After 10 days on the second follow-up, she presented to the clinic with a persistent complaint of abdominal pain over the right upper quadrant with a further resolution of herpetic skin lesion than the previous consultation. Ultrasonography of the abdomen, 2D echocardiography, and cardiac biomarkers were normal. She was continued on symptomatic care with pregabalin and amitriptyline and had become asymptomatic at day 46th of onset of COVID-19.

## Discussion

Emerging evidence suggests that COVID-19 not only affects the respiratory system but can affect various organ systems including the skin. A wide range of dermatological signs and symptoms have been reported ranging from maculopapular eruptions, morbilliform rashes, urticaria, chickenpox‐like lesions, livedo reticularis, COVID toes, erythema multiforme, pityriasis rosea, and several other patterns [1].

Aging is known to be HZ's most important risk factor. After acquiring a VZV infection, the T-cell immunity level starts to decline with time resulting in a reduction of protection against HZ [2]. Moreover, immuno-compromised states that may arise due to immunosuppressive medication, HIV infection, malignancy-like lymphomas, and stress may create a low T-cell level environment and hence are known risk factors for HZ [3]. Our case is of a woman that had recently recovered from COVID-19 as evident from her negative RT-PCR for COVID-19 with negative immunoglobulin M (IgM) antibody and positive immunoglobulin G (IgG) antibody for COVID-19 on presentation with HZ. Her white blood cell (WBC) counts were within normal range and with no evidence of immunosuppression.

Reactivation of HZ is not frequent during an active COVID-19 infection, but few of the reported cases have raised the concern of the possible association between the two. A case report of two cases demonstrated HZ reactivation preceding the emergence of respiratory symptoms in COVID-19 patients [4]. Moreover, HZ may occur in entirely asymptomatic COVID-19 patients [5]. In one study, HZ was diagnosed seven days before COVID-19 was reported with RT-PCR and the severity was unusual within the first 12 hours, probably due to timely initiation of therapy, which was attributed to exaggerated inflammatory response at dorsal root ganglion in the setting of SARS-CoV-2, a known precipitating factor triggering a strong host response [6]. In another case report, HZ presented two days after respiratory symptoms in an immunocompetent man [2]. The median time from COVID-19 to HZ diagnosis was 5.5 days, and four patients showed leukopenia at the time of diagnosis [7]. Prolonged dermatological manifestations in the form of non-itchy generalized maculopapular exanthem made a probable diagnosis of pityriasis rosea exanthem reported four weeks following the recovery of COVID-19 in a 12-year-old child [8].

In our case, a 62-year-old female presented with a typical unilateral painful, pruritic, vesicular rash over the dermatomes T-11-12, six weeks after she was diagnosed with COVID-19 and was treated for the same, recovering within two weeks. On presentation with the rash, the patient's blood counts were within normal range after recovering from COVID-19. Unlike other cases of HZ reported to date, this patient presented following the recovery of COVID-19 both clinically as well haematologically as evident from WBC count and IgM antibody, which were within the normal range at the time of presentation. Nevertheless, observations in patients with mild disease have illustrated significantly decreased T-cell and CD8 levels, indicating a possibility of SARS-CoV-2 directly infecting lymphocytes, which can eventually present in dysfunctional cells due to direct viral effects [9]. These outcomes create optimum habitat for HZ emergence during an active COVID-19 infection but do not explain the emergence of HZ after clinical and functional recovery.

## Conclusions

Previous studies have reported reactivation of HZ from three days prior to symptom onset of COVID-19 to 13 days after the diagnosis. There have been very few cases of HZ published in literature during convalescence or after recovery from COVID-19. To our knowledge, this is the first case of VZV reactivation after 20 days following recovery from COVID-19. Future studies need to focus on absolute lymphocytes, T-cells, and cytokine function in patients who present with reactivation of VZV during illness and convalescence from COVID-19 infection and its effects on cellular immunity with regards to its role in etiology, manifestation, and prognosis.
